# Pregnancy Outcomes Among Women With Sickle Cell Disease in Bahrain: A Case-Control Study

**DOI:** 10.7759/cureus.64995

**Published:** 2024-07-20

**Authors:** Batool J Marhoon, Aalaa A Marzooq, Heba A Alasfoor, Saeeda Albalooshi

**Affiliations:** 1 Obstetrics and Gynecology, Salmaniya Medical Complex, Manama, BHR; 2 Maternal-Fetal Medicine, American Mission Hospital, Manama, BHR

**Keywords:** maternal death, maternal morbidity, medical disorders in pregnancy, maternal and fetal outcome, bahrain, pregnant, sickle cell disease (scd)

## Abstract

Objectives

The study sought to evaluate and compare the maternal and fetal outcomes of pregnancy in women with sickle cell disease (SCD) versus healthy pregnant women in Bahrain. The objective was to update the available data in order to come up with a strategy to implement a multidisciplinary management program, which will enhance pregnancy outcomes for the SCD patient population.

Materials and methods

This retrospective case-control study was conducted in the Obstetrics and Gynecology Department at Salmaniya Medical Complex (SMC) in Bahrain. The study group consisted of all pregnant women with homozygous SCD (HbSS) who delivered at SMC between January 1, 2019, and December 31, 2021. The control group comprised pregnant women who delivered at SMC during the same period but did not have SCD or trait.

Data for the study were collected from the healthcare system records at SMC, specifically the I-Seha electronic medical record system and the labor room registry book. A thorough review and analysis of the data were conducted, encompassing 217 cases of SCD and 200 controls. The variables examined included nationality, age, gravidity, parity, gestational age, reason for admission, antenatal/postnatal complications (such as urinary tract infection, pneumonia, acute chest syndrome, thromboembolism, premature rupture of membranes, hypertension, pre-eclampsia, and intrauterine growth restriction), type of delivery, birth weight, newborn outcome, and postnatal complications.

Results

Pregnant women with SCD experienced significantly higher rates of antenatal hospitalization compared to controls - 69.6% were admitted at least twice versus only 16.5%. Vaso-occlusive crises were the primary reason for admission in over half of SCD patients, with 22.6% having one episode, 11.1% having two, and 20.3% having more than two during pregnancy. Low hemoglobin levels also necessitated admission in 11.1% of SCD women, while no controls required hospitalization for this.

The burden of maternal morbidity was substantially greater in the SCD group, with only 20.3% free of complications versus 94% in controls. SCD women had elevated rates of blood transfusions, acute chest syndrome, and urinary tract infections. Adverse pregnancy outcomes were also more common, including higher risks of preterm birth, low birth weight, and intrauterine growth restriction. Despite these increased maternal and fetal risks, there was no significant difference in the incidence of hypertensive disorders between groups. Interestingly, our data showed a significantly lower incidence of gestational diabetes in the SCD group compared to controls (8.3% vs. 18%). Tragically, one maternal death occurred in the SCD group, although the overall maternal mortality did not differ significantly.

Conclusion

SCD poses substantial risks for mother and fetus. Careful monitoring with a multidisciplinary team and patient education are crucial. Early detection can reduce morbidity and mortality. Further research is needed on interventions to improve outcomes.

## Introduction

Sickle cell disease (SCD) is one of the most prevalent genetic disorders globally. It is characterized by a broad spectrum of clinical severity, significant complications, and high rates of morbidity and mortality [1].

SCD is caused by a point mutation in the beta-globin gene on chromosome 11 that results in an amino acid change at position 6 that converts glutamic acid to valine, leading to increased polymerization of hemoglobin sickle (HbS) and red blood cell rigidity. This results in chronic hemolysis, nitric oxide depletion, and tissue hypoxia due to vascular occlusion [2]. Sickle cell syndrome is characterized by alternating recovery and crisis periods, including hemolytic, vaso-occlusive, aplastic, and sequestration crises. The most common sites of infarction are the spleen and bones, but there are no exceptional tissues [3].

The malaria hypothesis suggests that the gene responsible can reach high frequencies due to resistance to malaria in heterozygous carriers, a form of balanced selection. A Bayesian geostatistical analysis has mapped the global distribution of the HbS allele, supporting the malaria hypothesis, particularly in Africa [4]. While SCD is most common in people of African origin, it is also prevalent in Saudi Arabia, India, and Mediterranean regions [5].

In Bahrain, inherited hemoglobin disorders are highly prevalent. This is due to the historical prevalence of falciparum malaria in the country until 1970, which led to the widespread occurrence of malaria-associated genetic defects affecting red blood cells, including SCD, thalassemia, and glucose-6-phosphate dehydrogenase deficiency. A study conducted in Bahrain over a six-year period (1982-1987) in a hospital population covering 56,198 Bahraini citizens found that 2% of newborns had SCD, 18% were diagnosed with sickle cell trait, and 24% were carriers of thalassemia genes [6]. Hereditary anemia was ranked as the third most common diagnosis in 1990 at Salmaniya Medical Complex (SMC), the main hospital in Bahrain [7]. After the introduction of a national prevention program in the Kingdom of Bahrain, a recent study found that the incidence of SCD decreased to 0.4% [8].

Improvements in SCD care have enabled more individuals with the condition to reach reproductive age. The incidence of SCD among pregnant women in Bahrain has declined to 0.55% in recent years, down from higher rates of 0.8% and 0.7% reported in earlier studies [9-10].

Limited research has been conducted on pregnancy outcomes in this high-risk population within the local context. Prior Bahraini studies have consistently demonstrated increased feto-maternal morbidity and mortality among pregnant women with SCD compared to healthy controls. One of the latest studies from Bahrain analyzed pregnancy outcomes in women with SCD who gave birth more than 10 years ago (2011-2012). This study showed that patients with SCD required significantly more hospital admissions compared to controls. The majority (78.4%) of patients with sickle cell anemia required hospitalization during pregnancy, compared to 37.4% of controls. The difference was statistically significant. Approximately 65% of patients with SCD were hospitalized for vaso-occlusive crisis and 10% for hemolytic crisis [11]. A study conducted by Yu et al. found that sickling crisis required admission in 47% of the antenatal patients and more frequently in the third trimester [12].

A study conducted at the SMC in Bahrain in 2014 analyzed all maternal deaths reported in Bahrain between 1977 and 2012. Bahrain recorded 122 maternal deaths between 1977 and 2012, and 30% had SCD. This study showed a significant reduction in the total maternal mortality ratio (MMR). The most important causes of maternal death in SCD were embolism (35%), sepsis (24%), hemorrhage (16%), and acute chest syndrome (13.5%). Bahrain had a significant decline in MMR over the years, but unfortunately no significant decline among mothers with SCD [13].

Given the paucity of up-to-date, locally relevant data, this study aimed to assess maternal and fetal prognosis in pregnant women with SCD in Bahrain. The objective was to generate updated evidence to develop a multidisciplinary management program to enhance pregnancy outcomes for this vulnerable patient population.

## Materials and methods

Study design and setting

This retrospective case-control study was conducted at SMC, the premier government hospital in Bahrain and a distinguished referral center for high-risk pregnancies, making it an optimal setting for our investigation. The study population was identified from the labor room registry, a comprehensive record of all deliveries at SMC occurring after 22 weeks of gestation, in accordance with the viability criteria established by our local center.

Study participants

The case group included pregnant women with a confirmed diagnosis of homozygous SCD (HbSS) who delivered at SMC between January 1, 2019, and December 31, 2021. The diagnosis was determined through hemoglobin electrophoresis or genetic testing. A total of 217 HbSS cases were identified, excluding individuals with sickle cell trait or other SCD genotypes (e.g., HbSC and HbSD). The diagnosis was confirmed by reviewing hemoglobin test results conducted during pregnancy or before conception. In cases where the test results were unavailable, the diagnosis relied on documented physician notes or the presence of relevant ICD-10 codes (I-Seha electronic medical record system).

The control group was chosen by selecting patients who delivered immediately following a case of SCD, with the exclusion of individuals who had sickle cell trait or SCD. A total of 17 cases were excluded from the control group, resulting in a final sample size of 200 cases.

Data collection

Data were retrospectively collected from the labor room registry and electronic medical records (I-Seha) at SMC. Two independent researchers used a structured data collection form to systematically extract relevant information, including demographic characteristics, obstetric history, SCD-related complications, and maternal and neonatal outcomes. Discrepancies between the two researchers were resolved through discussion and consensus. In addition, a random sample of 10% of the medical records was re-reviewed, confirming the overall accuracy of the extracted data to be above 95%.

Statistical analysis

The collected data were analyzed using IBM SPSS Statistics for Windows, version 26.0 (released 2019, IBM Corp., Armonk, NY). Categorical variables were presented as frequencies and percentages, while continuous variables were reported as means and standard deviations. Independent-samples t-test was employed to compare means between the SCD and control groups. To assess the significance of differences in proportions between the SCD and control groups, the statistical tests employed were the chi-square test or, in cases where more than 20% of the expected counts were less than 5, the Fisher's exact test was utilized. A significance level of p < 0.05 was considered statistically significant for all tests performed.

Ethical approval

To ensure the ethical integrity of our study, we obtained ethical approval from the Research Committee for Government Hospitals in Bahrain, with approval serial number 79180922. This approval signifies that our study adheres to the established ethical guidelines and safeguards the rights and well-being of the participants involved.

## Results

The study included 217 women with SCD who delivered at SMC between January 1, 2019, and December 31, 2021. These SCD cases were compared to a control group of 200 women who delivered at SMC during the same timeframe, but did not have SCD or trait. The results will be presented in the following categories: characteristics of the SCD patients compared to the control group, admissions during pregnancy, antenatal complications, mode of delivery, maternal morbidities, neonatal outcomes, and mortality.

Characteristics of SCD patients as compared with the control group

Nationality

The vast majority of SCD patients were Bahraini (98.6%), in contrast to 59% of the control group. This difference was statistically significant, with a p-value less than 0.001 (Table 1).

**Table 1:** Sickle cell disease patients and control groups’ characteristics (categorical variables) P-value was computed by using Chi-square test. A p-value of less than 0.05 was considered statistically significant.

Variable		SCD (n = 217) n (%)	Control (n = 200) n (%)	P-value
Nationality	Bahraini	214 (98.6)	118 (59)	<0.001
	Non-Bahraini	3 (1.4)	82 (41)	
Age	≤25 years	54 (24.9)	52 (26)	0.794
	>25 years	163 (75.1)	148 (74)	
Gravidity	1	55 (25.3)	47 (23.5)	0.691
	2	64 (29.5)	58 (29)	
	3	51 (23.5)	42 (21)	
	>3	47 (21.7)	53 (26.5)	
Parity	0	67 (30.9)	63 (31.5)	0.864
	1	75 (34.6)	64 (32)	
	2	45 (20.7)	40 (20)	
	>3	30 (13.8)	33 (16.5)	
Gestational age	≤36 weeks	52 (24)	28 (14)	0.010
	>36 weeks	165 (76)	172 (86)	
Number of admissions	1	66 (30.4)	167 (83.5)	<0.001
	2	58 (26.7)	28 (14)	
	>2	93 (42.9)	5 (2.5)	
Number of VOC	0	100 (46.1)	------	------
	1	49 (22.6)	------	
	2	24 (11.1)	------	
	>2	44 (20.3)	------	
Birth weight	<2.5 kg	52 (24)	27 (13.5)	0.006
	≥2.5 kg	165 (76)	173 (86.5)	

Maternal Age

The mean maternal age at delivery was 29.82 ± 5.28 years for the SCD patients and 29.58 ± 5.67 years for the control group (Table 2). Fifty-four SCD patients (24.9%) and 52 control group women (26%) were aged 25 years or younger at the time of delivery. The difference between the two groups was not statistically significant (Table 1).

Gravidity and Parity

The mean parity was 1.23 ± 1.17 for the SCD group compared to 1.38 ± 1.43 for the control group (Table 2). Fifty-five SCD patients (25.3%) were primigravid, while 47 control group women (23.5%) were primigravid. The remaining patients were multigravida (74.7% in the SCD group, 76.5% in the control group). The difference in gravidity between the two groups was not statistically significant (Table 1).

Gestational Age

The mean gestational age at delivery was 36.97 ± 1.92 weeks for the SCD group, compared to 38.07 ± 2.5 weeks for the control group (Table 2). Fifty two SCD patients (24%) delivered at 36 weeks of gestation or earlier, while 28 control group women (14%) delivered at 36 weeks or earlier. This difference was statistically significant, with a p-value of 0.010 (Table 1).

**Table 2:** Sickle cell disease patients and control groups’ characteristics (quantitative variables) The p-value was computed by using independent-samples t-test; a p-value of less than 0.05 was considered statistically significant. VOC: vaso-occlusive crises

Variable		Mean +/- SD	95% CI lower	95% CI upper	P-value
Age	SCD	29.82 +/- 5.28	29.11	30.52	0.654
	Control	29.58 +/- 5.67	28.78	30.37	
Gravidity	SCD	2.60 + /- 1.53	2.40	2.81	0.233
	Control	2.79 +/- 1.66	2.56	3.02	
Parity	SCD	1.23 +/- 1.17	1.07	1.39	0.261
	Control	1.38 +/- 1.43	1.18	1.57	
Gestational age	SCD	36.97 +/- 1.92	36.71	37.23	<0.001
	Control	38.07 +/- 2.50	37.72	38.42	
Number of admissions	SCD	2.93 +/- 2.50	2.59	3.26	<0.001
	Control	1.20 +/- 0.48	1.13	1.26	
Number of VOC	SCD	1.47 +/- 2.47	1.14	1.80	------
	Control	-----------	------	------	
Birth weight	SCD	2.81 +/- 0.52	2.74	2.88	<0.001
	Control	3.07 +/- 0.65	2.98	3.16	

Admissions during pregnancy

Number of Admissions

The majority of SCD patients had multiple antenatal admissions, with 26.7% admitted twice and 42.9% admitted more than twice. Sixty six out of 217 SCD patients (30.4%) were admitted only once during pregnancy. By contrast, 167 out of 200 women in the control group (83.5%) were admitted just once antenatally, with 14% having two admissions and 2.5% admitted more than two times (Table 1). The mean number of admissions was 2.93 ± 2.5 for the SCD group, compared to 1.2 ± 0.48 for the control group. This difference was statistically significant (p < 0.001).

Reasons of Admissions

Vaso-occlusive crises (VOC) were the primary reason for hospitalization in the SCD group, with 53% of SCD patients admitted for this reason (Table 3). The mean number of VOC episodes was 1.47 ± 2.47 (Table 2), with 22.6% of SCD patients experiencing one VOC, 11.1% having two VOCs, and 20.3% having more than two VOC episodes (Table 1).

**Table 3:** Reason of admission in the SCD and control groups The p-value was computed by using Chi-square test; a p-value of less than 0.05 was considered statistically significant. IOL: induction of labor, VOC: vaso-occlusive crises, LSCS : lower-segment cesarean section, PROM: premature rupture of membranes, HB: hemoglobin, UTI: urinary tract infection, PPROM: preterm premature rupture of membranes, SCD: sickle cell disease

Reason of admission	SCD (n = 217) n (%)	Control (n = 200) n (%)	P-value
IOL	61 (28.1)	57 (28.5)	0.930
VOC	115 (53)	------	------
Labor	40 (18.4)	73 (36.5)	<0.001
Elective LSCS	29 (13.4)	26 (13)	0.913
PROM	13 (6)	20 (10)	0.130
Low HB	24 (11.1)	0 (0)	<0.001
UTI	14 (6.5)	2 (1)	0.004
PPROM	6 (2.8)	8 (4)	0.484
Others	28 (12.9)	23 (11.5)	0.662

A percentage (18.4%) of the SCD group were admitted for spontaneous labor, compared to 36.5% of the control group (Table 3). Low hemoglobin levels were a reason for admission in 11.1% of the SCD group, but in none of the control group.

Urinary tract infections (UTI) requiring admission were seen in 6.5% of the SCD group, versus only 1% in the control group. These differences in admission reasons between the two groups were statistically significant (Table 3).

Other reasons for admission, such as elective lower-segment cesarean sections (LSCS), induction of labor (IOL), premature rupture of membranes (PROM)/preterm premature rupture of membranes (PPROM), and miscellaneous causes, did not show statistically significant differences between the groups (Table 3).

Antenatal complications

UTI

The study found that 34.1% of SCD women experienced UTIs during pregnancy, compared to 13% of the control group. This difference was statistically significant (p < 0.001) (Table 4). As mentioned previously, 6.5% of the SCD patients with UTI required hospitalization, compared to only 1% in the control group (Table 3).

**Table 4:** Antenatal complications in the SCD and control groups The p-value was computed by using Chi-square test or Fisher’s exact test; a p-value of less than 0.05 was considered statistically significant. UTI: urinary tract infection, HTN: hypertension, PIH: pregnancy-induced hypertension, OBS: obstetric, PROM: premature rupture of membranes, APH: antepartum hemorrhage, PPROM: premature preterm rupture of membranes, IUGR: intrauterine growth restriction, DM: diabetes mellitus, GDM: gestational diabetes, SCD: sickle cell disease

Antenatal complications	SCD (n = 217) n (%)	Control (n = 200) n (%)	P-value
UTI	74 (34.1)	26 (13)	<0.001
HTN	1 (0.5)	3 (1.5)	0.354
Preeclampsia	8 (3.7)	7 (3.5)	0.919
Eclampsia	1 (0.5)	0 (0)	1.000
PIH	11 (5.1)	14 (7)	0.407
OBS cholestasis	7 (3.2)	3 (1.5)	0.341
PROM	16 (7.4)	21 (10.5)	0.262
APH	5 (2.3)	9 (4.5)	0.210
PPROM	9 (4.1)	9 (4.5)	0.860
IUGR	33 (15.2)	11 (5.5)	0.001
DM	4 (1.8)	5 (2.5)	0.743
GDM	18 (8.3)	36 (18)	0.003

IUGR

In the SCD group, 33 out of 217 patients (15.2%) had pregnancies complicated by intrauterine growth restriction (IUGR), compared to 11 out of 200 (5.5%) in the control group. This difference was statistically significant (p = 0.001) (Table 4).

DM and GDM

Four patients (1.8%) in the SCD group were diagnosed with diabetes mellitus (DM), compared to five patients (2.5%) in the control group. This difference was not statistically significant. However, gestational diabetes (GDM) was more common in the control group, affecting 36 out of 200 patients (18%), compared to 18 out of 217 (8.3%) in the SCD group. This difference was statistically significant (p = 0.003) (Table 4).

Hypertensive Disorders (Essential HTN, PIH, Preeclampsia, and Eclampsia)

One patient (0.5%) in the SCD group had essential hypertension (HTN), 11 (5.1%) had pregnancy-induced hypertension, eight (3.7%) had preeclampsia, and one (0.5%) progressed to eclampsia. In the control group, three patients (1.5%) had essential hypertension, 14 (7%) developed pregnancy-induced hypertension (PIH), and seven (3.5%) had preeclampsia, with no cases of eclampsia. The differences between the two groups were not statistically significant (Table 4).

Other antenatal complications including PROM, PPROM, APH, and obstetric cholestasis showed no significant statistical difference between SCD and control group (Table 4).

Mode of delivery

Regarding the mode of delivery, the study found the following: In the SCD group, 111 patients (51.2%) had vaginal deliveries. Fifty nine patients (27.2%) required emergency cesarean sections, and 39 patients (18%) had elective cesarean sections. In addition, eight patients (3.7%) underwent vacuum-assisted vaginal deliveries, and no patients had assisted breech deliveries.

In the control group, the distribution of delivery modes was similar. One hundred fifteen patients (57.5%) had vaginal deliveries. Fifty two patients (26%) required emergency cesarean sections, and 26 patients (13%) had elective cesarean sections. Six patients (3%) underwent vacuum-assisted vaginal deliveries, and one patient (0.5%) had an assisted breech delivery.

Importantly, the statistical analysis showed no significant differences in the modes of delivery between the two groups (Table 5).

**Table 5:** Mode of delivery in the SCD and control groups The p-value was computed by using Chi-square test or Fisher’s exact test; a p-value of less than 0.05 was considered statistically significant. LSCS: lower-segment cesarean section, SCD: sickle cell disease

Mode of delivery	SCD (n = 217) n (%)	Control (n = 200) n (%)	P-value
Vaginal delivery	111 (51.2)	115 (57.5)	0.194
Emergency LSCS	59 (27.2)	52 (26)	0.784
Elective LSCS	39 (18)	26 (13)	0.162
Vacuum-assisted vaginal delivery	8 (3.7)	6 (3)	0.697
Assisted breech delivery	0 (0)	1 (0.5)	0.480

Maternal morbidities

The prevalence of maternal morbidities and complications was significantly higher among the SCD group compared to the control group. Only 20.3% of SCD women were free of morbidities and complications, whereas 94% of the control group had no complications. SCD women had substantially higher rates of certain complications, including blood transfusion (67.7% vs. 2.5%), exchange blood transfusions (26.7% vs. 0%), and acute chest syndrome (5.5% vs. 0%). In addition, there were other less common complications that were combined and labeled as "others," such as extended perineal tears, cervical tears, wound hematomas, urinary retention, cellulitis, osteomyelitis, acute cholecystitis, postpartum cardiomyopathy, uterine rupture, hysterectomy, and disseminated intravascular coagulation. The rate of these "other" complications was 8.8% in the SCD group compared to 1.5% in the control group, a statistically significant difference.

By contrast, other studied complications showed no significant differences between the SCD and control groups, including postpartum hemorrhage (PPH) (3.2% vs. 1.5%), pneumonia (2.8% vs. 0.5%), pulmonary embolism (PE) (1.8% vs. 0%), and wound infection (0.5% vs. 1%) (Table 6).

**Table 6:** Morbidity in the SCD and control groups The p-value was computed by using Chi-square test or Fisher’s exact test; a p-value of less than 0.05 was considered statistically significant. PE: pulmonary embolism, PPH: postpartum hemorrhage, SCD: sickle cell disease

Morbidity	SCD (n = 217) n (%)	Control (n = 200) n (%)	P-value^1^
No morbidity	44 (20.3)	188 (94)	<0.001
Blood transfusion	147 (67.7)	5 (2.5)	<0.001
Exchange blood transfusion	58 (26.7)	0 (0)	<0.001
Acute chest syndrome	12 (5.5)	0 (0)	0.001
PPH	7 (3.2)	3 (1.5)	0.342
Pneumonia	6 (2.8)	1 (0.5)	0.124
PE	4 (1.8)	0 (0)	0.124
Wound infection	1 (0.5)	2 (1)	0.609
Others	19 (8.8)	3 (1.5)	0.001

Neonatal outcome

Birth Weight

Fetal birth weight was significantly lower in the SCD group compared to the control group (2.81 ± 0.52 kg vs. 3.07 ± 0.65 kg) (Table 2). In the SCD group, 24% of mothers (52 patients) had babies weighing less than 2.5 kg, while 76% (165 patients) had babies weighing 2.5 kg or more (Table 1). By contrast, 13.5% of the control group (27 patients) had babies weighing less than 2.5 kg, and 86.5% (173 patients) had babies weighing 2.5 kg or more (Table 1).

Apgar Score

There was no significant difference in the Apgar scores between the two groups. In the SCD group, 93.1% and 96.3% of babies scored 7 or higher at one minute and five minutes of life, respectively, compared to 95% and 98.5% in the control group. Conversely, 2.8% and 2.3% of the babies in the SCD group had Apgar scores of 3 or less at one minute and five minutes, respectively, compared to 2% and 1% in the control group (Table 7).

**Table 7:** Apgar score at one and five minutes of the SCD and control groups The p-value was computed using the Chi-square test or Fisher’s exact test; a p-value of less than 0.05 was considered statistically significant. Min: minute, SCD: sickle cell disease

Apgar score		SCD (n = 217) n (%)	Control (n = 200) n (%)	P-value
Apgar score at 1 min	0–3	6 (2.8)	4 (2)	0.753
	4–6	9 (4.1)	6 (3)	0.530
	7–10	202 (93.1)	190 (95)	0.411
Apgar score at 5 min	0–3	5 (2.3)	2 (1)	0.452
	4–6	3 (1.4)	1 (0.5)	0.624
	7–10	209 (96.3)	197 (98.5)	0.164

Infants in the control group had significantly lower rates of morbidities and complications compared to the SCD group. Specifically, 80.5% of control infants had no abnormalities detected, compared to 56.7% of infants in the SCD group. Infants born to mothers with SCD had a significantly higher prevalence of neonatal jaundice (35% vs. 12.5%) and neonatal abstinence syndrome (4.6% vs. 0%) compared to the control group.

For other studied complications, there were no significant differences between the SCD and control groups, including congenital heart disease (3.2% vs. 2.5%), fetal/neonatal death (3.2% vs. 1.5%), hypoxic ischemic encephalopathy (HIE) (3.2% vs. 1%), intraventricular hemorrhage (IVH) (1.8% vs. 1.5%), and congenital anomalies (0.9% vs. 1%).

There were also some less common complications that were grouped together and labeled as "others," such as hypoglycemia, hemoglobinopathies, meconium plug syndrome, meconium aspiration syndrome, Erb's palsy, anemia, and infections requiring blood transfusion. The rate of these "other" complications was 6.9% in the SCD group and 5.5% in the control group, a difference that was not statistically significant (Table 8).

**Table 8:** Neonatal outcome in the SCD and control groups The p-value was computed by using the Chi-square test or Fisher’s exact test; a p-value of less than 0.05 was considered statistically significant. HIE: hypoxic-ischemic encephalopathy, IVH: intraventricular hemorrhage

Neonatal outcome	SCD (n = 217) n (%)	Control (n = 200) n (%)	P-value
No abnormality detected	123 (56.7)	161 (80.5)	<0.001
Neonatal jaundice	76 (35)	25 (12.5)	<0.001
Congenital heart disease	7 (3.2)	5 (2.5)	0.658
Fetal / neonatal death	7 (3.2)	3 (1.5)	0.342
Neonatal abstinence syndrome	10 (4.6)	0 (0)	0.002
HIE	7 (3.2)	2 (1)	0.178
Chromosomal aberration	4 (1.8)	4 (2)	1.000
IVH	4 (1.8)	3 (1.5)	1.000
Congenital anomalies	2 (0.9)	2 (1)	1.000
Others	15 (6.9)	11 (5.5)	0.551

Mortality

Maternal Mortality

Tragically, there was one maternal mortality in the SCD group. This patient developed a pulmonary embolism during her hospital stay and went into labor at 31 weeks of gestation, which was complicated by uterine rupture due to her previous history of two cesarean sections. Her emergency life-saving cesarean section ended up with hysterectomy followed by maternal death. The incidence of maternal mortality in the SCD group was 0.5%, compared to 0% in the control group, but this difference was not statistically significant (Table 9).

**Table 9:** Mortality in the SCD and control groups The p-value was computed by using Fisher’s Exact test; a p-value of less than 0.05 was considered statistically significant. IUFD: intrauterine fetal death, SCD: sickle cell disease

Mortality	SCD (n = 217) n (%)	Control (n = 200) n (%)	P-value
No mortality	210 (96.8)	197 (98.5)	0.342
Neonatal death	4 (1.8)	1 (0.5)	0.374
Stillbirth	1 (0.5)	2 (1)	0.609
IUFD	2 (0.9)	0 (0)	0.500
Maternal death	1 (0.5)	0 (0)	1.000

Perinatal and Neonatal Mortality

There was no significant difference in the incidence of perinatal or neonatal mortality between the SCD and control groups. The neonatal mortality rate was 1.8% in the SCD group, compared to 0.5% in the control group. The rates of stillbirth and intrauterine fetal death were 0.5% and 0.9%, respectively, in the SCD group, compared to 1% and 0% in the control group (Table 9).

## Discussion

Nowadays, the life expectancy of people with SCD has increased due to improvements in healthcare facilities, antibiotic prophylaxis, vaccination, and the availability of medications such as hydroxyurea. More women with SCD are now reaching their childbearing age and expressing their desire to have children [14]. Pregnancies in women with sickle cell anemia can be complicated by the high risk of adverse outcomes for both the mother and the newborn. In patients with SCD, the physiological changes of pregnancy, such as increased metabolic demands, increased blood viscosity, and hypercoagulability, are exacerbated, leading to a higher incidence of complications [15-16].

In the study, SCD patients had significantly more antenatal hospital admissions, with 69.6% admitted at least twice, compared to 16.5% in the control group. The reasons for hospitalization varied in the SCD group, with VOCs being the most common; 53% of women with SCD were admitted due to VOCs. Notably, 22.6% of SCD patients had one VOC episode during pregnancy, 11.1% had two, and 20.3% had more than two. In addition, 11.1% of the SCD group were admitted due to low hemoglobin levels, while none in the control group were. These findings are consistent with a study conducted in the United States, as well as another study by Rajab KE, which found that pregnancy can worsen episodes of pain crises and increase hospitalization rate. Most of the admissions during the antenatal period were due to pain crises [15,17]. A recent study on SCD pregnant women in Bahrain had almost the same findings, where 78.4% of SCD patients were admitted antenatally compared with 37.4% of the controls. VOCs accounted for 65% of antenatal admissions of SCD patients, followed by hemolytic crises (10%) [11]. However, in a 10-year retrospective study in Saudi Arabia, 86.2% of maternal complications were anemia, followed by sickle cell crisis (64.8%) [3].

The prevalence of maternal morbidities and complications was significantly higher among the SCD group across most of the studied morbidities and complications; only 20.3% of SCD women were free of morbidities and complications compared with 94% of the controls. SCD women had significantly higher prevalence rates of blood transfusion, exchange blood transfusions, and acute chest syndrome compared with the controls. This is consistent with the results of a systematic review and meta-analysis of 47 studies, which showed a statistically significant increased risk of blood transfusion in pregnant women in seven studies (relative risk (RR): 13, 95% confidence interval (CI): 6.2, 26) and a statistically increased risk of acute chest syndrome in pregnant women with SCD based on four studies (RR: 33, 95% CI: 7.5, 137.5) [18].

The literature and meta-analysis found a statistically significant increased risk of UTIs in pregnant women with SCD based on seven studies (RR: 2.1, 95% CI: 1.8, 2.4) [18]. This was observed in our data, where SCD women had significantly higher rates of UTI during their pregnancy than controls. Compared to a study done in Bahrain among SCD patients who delivered between 2011 and 2012, the incidence of UTI in SCD patients increased from 11.6% to 34.1% [11]. The increased risk of infections can be attributed to auto-splenectomy in SCD patients, leading to defective splenic functions, in addition to the immunocompromised status of pregnancy.

In addition to the elevated risk of medical comorbidities, pregnancy-related complications tend to be higher in patients with SCD. Consistent with other published studies [10-11,15,17-23], we found a significantly increased risk of preterm delivery, low birth weight, and intrauterine growth restriction in the SCD group. Low maternal blood oxygen levels in anemia can affect placental circulation, increasing the risk of intrauterine growth restriction. Preterm birth can be explained by several potential causes, such as infection during pregnancy, a known cause of spontaneous preterm birth. In addition, the frequent maternal and fetal comorbidities associated with SCD patients may necessitate early termination of pregnancy [23]. The high risk of prematurity itself can explain the increased incidence of low birth weight among women with SCD. Other risk factors for low birth weight include low maternal weight, a prior history of preeclampsia and a history of severe anemia [24].

Our study revealed no significant difference in the incidence of hypertensive disorders between the two groups. This aligns with the results of three previous studies conducted in Bahrain [9,11,17]. However, this contrasts with many other studies that have found significantly increased risks of pregnancy-induced hypertension and preeclampsia in women with SCD [10,14-15,18-22,25]. The literature and published studies have found no significant association between SCD and gestational diabetes. Interestingly, our data showed that the incidence of gestational DM was significantly lower in the SCD group than in the control group (8.3% vs. 18%).

Furthermore, our results found no significant difference between the two groups in the incidence of all types of delivery, including cesarean section, which is consistent with the results of the recent local study by Al-Jufairi et al. [11]. By contrast, other studies have reported higher cesarean delivery rates in patients with SCD [14-15,18,23,25]. The increased rates may be explained by the high frequency of pregnancy complications and comorbidities in SCD patients. In addition, some centers have a policy of short trial labor and early surgical intervention for patients with SCD at the first sign of fetal distress [26].

Morbidity and complications were significantly lower in infants in the control group; no abnormalities were detected in 80.5% of the infants compared with 56.7% in the SCD group. Infants in the SCD group had significantly higher rates of neonatal jaundice and neonatal abstinence syndrome compared to controls. In Ghana, NICU admissions due to fetal complications were higher in the SCD group [25]. According to six studies, pregnant women with SCD had a statistically significant higher neonatal death risk (RR: 2.2, 95% CI: 1.4, 4.5) [18]. However, SCD did not increase the risk of fetal death in the local study by Al-Jufairi et al. [11] or in our study, which can be explained by improvements in neonatal care.

In 2014, a study at SMC in Bahrain reviewed 122 maternal deaths from 1977 to 2012 and found that SCD was the main cause of maternal mortality, accounting for 30% of maternal deaths [13]. Unfortunately, there was one maternal mortality among the patients in the SCD group in our study. Our study, along with the latest local study conducted by Al-Jufairi et al. [11], found no significant difference in maternal deaths between the SCD and control groups. Many other studies, however, have found that maternal mortality was significantly increased in SCD patients, indicating the significant compromise in the health of SCD women during their pregnancies [14,18-20,25]. Most of maternal mortality cases in individuals with SCD occur abruptly. Acute respiratory failure is frequently observed and is often come with acute chest syndrome or pulmonary embolism [16,27]. In a study by Villiers et al., thromboembolic events were the leading cause of maternal death among women with SCD [15].

In comparison with the latest study on pregnancy outcomes in SCD patients in Bahrain [11], SCD continued to cause significantly higher morbidity and mortality on both the mother and fetus.

Limitations

One potential limitation of this study was the relatively small sample size of 217 SCD cases and 200 controls. While substantial, a larger, multicenter study could provide more statistical power and robust estimates of the effects. In addition, as a single-center study conducted at the major SCD treatment facility in Bahrain, the findings may have limited generalizability to other healthcare settings with different resources and patient populations.

Furthermore, as a retrospective case-control design, the analysis relied on data previously recorded in medical charts and registries, which may have contained incomplete or inaccurate information. Prospective data collection with standardized protocols could enhance the reliability of the data. Finally, the study was limited to short-term maternal and fetal outcomes during pregnancy and delivery, without longer-term follow-up of mothers and infants. Extending the evaluation period could offer important insights into the sustained impact of SCD on health and development.

While this study provides valuable insights, addressing these limitations in future research could further strengthen the evidence and guide the development of more effective interventions for this high-risk population.

## Conclusions

SCD remains a significant health concern for pregnant women and their developing fetuses. Regular monitoring and screening by a multidisciplinary team of hematologists, obstetricians, and pediatricians is crucial for managing associated complications. In addition, providing patient education and facilitating open communication is key. This comprehensive approach enables prompt detection and appropriate management of acute issues, thereby reducing mortality and morbidity rates. Further research is needed to develop more effective interventions and preventive measures that can improve outcomes for this patient population.
